# Adipose Tissue-Derived Therapies for Osteoarthritis: Multifaceted Mechanisms and Clinical Prospects

**DOI:** 10.3390/cells14090669

**Published:** 2025-05-02

**Authors:** Hanwen Zhang, Oliver Felthaus, Lukas Prantl

**Affiliations:** Department of Plastic, Hand and Reconstructive Surgery, University Hospital Regensburg, Franz-Josef-Strauß Allee 11, 93053 Regensburg, Germany

**Keywords:** adipose tissue, osteoarthritis, clinical therapy

## Abstract

Osteoarthritis (OA) is a degenerative joint disease that significantly impacts quality of life and poses a growing economic burden. Adipose tissue-derived therapies, including both cell-based and cell-free products, have shown promising potential in promoting cartilage repair, modulating inflammation, and improving joint function. Recent studies and clinical trials have demonstrated their regenerative effects, highlighting their feasibility as a novel treatment approach for OA. This review summarises the therapeutic mechanisms and latest advancements in adipose tissue-derived therapies, providing insights into their clinical applications and future prospects.

## 1. Introduction

According to the latest Global Burden of Disease (GBD) update, osteoarthritis (OA) has affected 7.6% of the world’s population (approximately 595 million people) [1]. This rising incidence imposes a heavy physical, social, and economic burden on patients [2]. Currently, clinical improvement is mainly achieved through pharmacological interventions (e.g., nonsteroidal anti-inflammatory drugs (NSAIDs), intra-articular injections (hyaluronic acid, corticosteroids, and platelet-rich plasma), and surgical means (cartilage grafting, arthroplasty, etc.) [3,4,5,6,7]. However, these treatments do not reverse the pathophysiology of OA and are often limited by drug addiction, complications (e.g., gastrointestinal or cardiovascular issues), and risks of invasive procedures [8,9,10]. Therefore, there is an urgent need to explore and develop innovative therapies that can target the pathophysiology of OA.

Recent studies have shown that adipose tissue and its derivatives have become a therapeutic tool of interest in the field of regenerative medicine due to their abundant sources, easy accessibility, and less ethical constraints [11,12]. Such derivatives include both cellular products such as microfragmented adipose tissue (MFAT), adipose tissue stromal vascular rich fraction (SVF), dedifferentiated adipocytes (DFAT), adipose tissue-derived stem cells (ADSCs), and non-cellular products such as adipose-derived stem cell extracellular vesicles (ADSC-EVs) and cell-free fat extracts (CEFFEs) [13,14,15,16,17]. Different derivatives have their own advantages and may reduce OA symptoms through multiple mechanisms (modulating the inflammatory microenvironment, promoting cartilage repair, etc.). Both preclinical and clinical studies have shown that adipose tissue-derived therapies have great potential to promote cartilage regeneration, reduce inflammation, and improve joint function.

With this in mind, the aim of this review is to summarise the latest advances in the use of adipose tissue and its derivatives for the treatment of OA. We will focus on their biological mechanisms, efficacy and clinical feasibility, and analyse the advantages and limitations of each approach. By integrating the latest research findings, this review seeks to provide researchers and clinicians in the field with a comprehensive view of the potential value of adipose-derived therapies in the treatment of OA, and to further help meet the unmet clinical needs.

## 2. Microfragmented Adipose Tissue in OA

**Figure 1:**
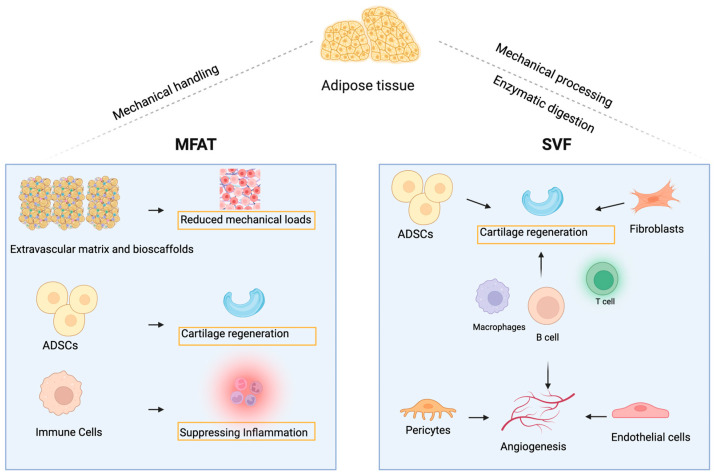
Mechanisms of action of MFAT and SVF within the OA joint. MFAT provides a biological scaffold to buffer joint pressure and support cartilage regeneration, while SVF delivers active cells to suppress inflammation and promote cartilage repair.

Most MFAT is obtained from adipose tissue by conventional liposuction and then placed in a mechanical treatment cassette, such as Lipogems^®^, for gentle mechanical treatment and filtration to remove erythrocytes and oil residues [18]. However, it has been shown that adipose tissue microfragmentation can also be performed without specialised equipment [19]. This cell-enriched lipotransfer (CELT) has already shown very good results in soft tissue augmentation. MFAT, as a purely physical treatment, is effective in avoiding issues such as cell contamination and biosafety compared to enzymatic methods [20,21]. It has been shown that MFAT is enriched with ADSCs and has a high angiogenic potential [18,22]. Also, MFAT injected into the joints may provide a bioscaffolding function and reduce the mechanical load on cartilage [23]. In an OA rat model, intra-articular injection of MFAT enhanced cartilage repair and chondrocyte migration. Researchers have hypothesised that MFAT acts as a ‘natural scaffold’ that secretes growth factors and extracellular vesicles (EVs) in the joint environment, further promoting cartilage repair [24]. In another study, MFAT was found to reverse tumour necrosis factor-α (TNF-α)-induced inflammation and regulate the target gene KLHL29 by upregulating the expression of miR-92a-3p, thereby improving the biological function of OA synoviocytes [25]. Figure 1 briefly depicts the mechanism of action of MFAT.

In recent years, MFAT for OA has achieved good results in clinical practice (Table 1). In patients with knee OA, Boric et al. observed a significant increase in glycosaminoglycan content in articular cartilage after 24 months of intra-articular MFAT injection [26]. Another one-year follow-up study of 110 patients with knee OA showed that intra-articular injection of MFAT significantly improved pain (visual analogue scale, VAS) scores, joint function, and quality of life [27]. However, some authors have reported that although symptomatic relief was achieved early after MFAT injection, only about 45% of patients maintained sustained improvement after 12 months [28]. On the other hand, Gobbi et al. found that MFAT had a sustained effect on joint function and quality of life in OA patients with Kellgren–Lawrence (KL) grades of II-IV at a 2-year follow-up [29]. Russo et al. pointed out that the higher the KL grade, the better the clinical outcome. MFAT can also be used as an alternative for patients who are unable or unwilling to undergo arthroplasty [30]. Onorato et al. further demonstrated that MFAT still has a positive long-term effect in patients with early OA at a 4-year follow-up [13]. Additionally, in the latest randomised controlled trial (RCT) study, MFAT was shown to significantly improve the VAS, Knee Injury and Osteoarthritis Score (KOOS), and Western Ontario and McMaster Universities Osteoarthritis Index (WOMAC) scores compared to a control group with intra-articular steroid and saline injections [31].

In addition to the knee, MFAT also performs well in injection therapy for other joints, such as the ankle and the hip [32,33,34]. Some clinical studies have shown significant improvement in pain and functional scores in patients with mild to moderate shoulder OA after articular injection of MFAT [35,36]. However, in a study by Baria et al., the body mass index (BMI) was found to be negatively correlated with treatment outcome scores in a population of patients treated with MFAT [37]. This may be due to the fact that people with a high BMI have higher levels of pro-inflammatory cytokines (interleukin-1β (IL-1β), IL-6, etc.) within their fat, which leads to a decrease in the effectiveness of MFAT [38]. Overall, MFAT has demonstrated good safety and efficacy in clinical applications, but its specific mechanism still requires more in-depth research.

**Table 1:** Clinical studies of MFAT in the treatment of OA.

Time	Country	Patient Number	Position (KL)	Age (Mean, Years)	Follow-Up	Results	Complications	Reference
2017	Croatia	17 (12M/5F)	Knee OA (III-IV)	69 ± 12	12 months	↑ GAG ↓ VAS, CRP	None	[39]
2019	USA	35 (12M/23F)	Knee OA(I-IV)	63 ± 11	1.09 ± 0.49 year	↓ VAS ↑ KOOS, EQOL score	None	[40]
2019	Croatia	10	Knee OA(III-IV)	69 ± 12	24 months	↑ GAG	None	[26]
2020	UK	110 (60M/50F)	Knee OA(I-IV)	42–94	12 months	↓ VAS ↑ OKS, EQ-5D	None	[27]
2020	USA	25	GHJ OA (II-IV)	>40	12 months	↓ VAS, DASH ↑ GHJ space	None	[36]
2021	United Arab Emirates, USA, Italy	75 (34.7%M/65.3%F)	Knee OA (II-IV)	69.6	24 months	↓ VAS, ↑ KOOS—ADL, KOOS-Pain	Adipose tissue harvest site pain (49% of patients) and swelling/bruising (28% of patients); knee swelling (13% of patients)	[29]
2021	Belgium	64 (48.4%M/51.6%F)	Knee OA (I-IV)	54.2 ± 9.1	12 months	↓ VAS ↑ KOOS—ADL, QOL; EQ-5D	Knee pain, swelling, and stiffness (79% of patients); knee instability (2 patients) and calf muscle soreness (1 patient)	[28]
2021	UK	220 (125M/95F)	Knee OA (III-IV)	-	24 months	↑ EQ-5D, OKS	Adipose tissue harvest site pain and bleeding (6%, 4% of patients); Knee pain and swelling (14% of patients); severe reactions to injections (1 patient)	[41]
2022	Italy	202 (97M/105F)	Knee OA (I-IV)	54.0 ± 9.0	24.5 ± 9.6 months	↓ VAS ↑ KOOS	None	[42]
2022	Italy	53 (28M/25F)	Knee OA (I-IV)	54.5 ± 12.1	24 months	↓ VAS ↑ IKDC Subjective scoring, KOOS-Pain	Mild or moderate knee pain, joint swelling and/or effusion (10 patients), pain and oedema in the treated leg (1 patient)	[43]
2022	Italy	55 (22M/33F)	Hip OA (I-IV)	52.5 ± 10.9	35 ± 6 months	↑ OHS	Adipose tissue harvest site bruising (1 patient)	[33]
2023	UK	46 (28M/18F) 13 (4M/9F)	Knee OA (I-IV) GHJ OA (III-IV)	66.9 ± 1.0 64.2 ± 2.4	52 weeks	↓ VAS, DASH ↑ OKS, Lysholm score	None	[44]
2023	China	20 (8M/12F)	Knee OA (I-IV)	54.63 ± 3.90	18 months	↓ VAS, WOMAC score (%) ↑ HSS, KSS, knee oedema	None	[45]
2024	USA	26 (8M/18F)	Knee OA (I-IV)	56.7 ± 7.8	12 months	↓ VAS ↑ KOOS, Tegner score	None	[46]
2025	USA	23 (15M/8F)	Knee OA (I-IV)	62.6	12 months	↓ VAS, WOMAC score (%) ↑ KOOS	Adipose tissue harvest site morbidity of mild pain and ecchymosis (minimal patients)	[31]

M: male; F: female; KL: Kellgren–Lawrence; GAG: glycosaminoglycan; ADL, activity of daily living; QOL, quality of life; EQOL, Emory Quality of Life; OKS, Oxford Knee Score; EQ-5D, EuroQuol 5D; GHJ, glenohumeral joint; DASH, Disabilities of the Arm, Shoulder and Hand; IKDC, International Knee Documentation Committee; OHS, Oxford Hip Score; HSS, Hospital for Special Surgery score; KSS, The Knee Society Score; WOMAC, Western Ontario and McMaster Universities Osteoarthritis Index; VAS, visual analogue scale; KOOS, Knee injury and Osteoarthritis Outcome Score.

## 3. Adipose Tissue Stromal Vascular Rich Fraction in OA

SVF is a mixed cell population that can be obtained by mechanical treatment or enzymatic digestion of adipose tissue. SVF mainly comprises vascular-derived cells such as ADSCs, macrophages, fibroblasts, endothelial cells, smooth muscle cells, and pericytes [21]. It has been found that SVF and ADSCs have similar effects in the treatment of a rat OA model [47] (Figure 1). Anjiki et al. found that SVF was mainly enriched with M2-type macrophages. SVF treatment enhances chondrocytes’ collagen II and SOX9 expression, thereby maintaining chondrocyte homeostasis [48]. In a further study, it was found that M2 macrophages in SVF were paracrine-regulated via the Transforming growth factor-β (TGF-β)-induced Smad2/3 phosphorylation pathway to support cartilage regeneration [49]. Recently, Onoi et al. showed that M2 macrophages in SVF can act directly on OA tissue to promote the secretion of growth factors and chondroprotective cytokines [50].

The results of several clinical studies have shown that SVF offers significant advantages in the treatment of OA (Table 2). A double-blind, randomised, self-controlled trial showed that patients receiving SVF injections had better WOMAC and VAS scores and knee mobility than hyaluronic acid controls, accompanied by better cartilage repair [51]. In another study, Tsubosaka et al. followed up on patients who received intra-articular knee injections of 2.5 × 10^7^ SVF cells and reported significant improvements in the WOMAC, VAS, and KOOS scores at 6 and 12 months postoperatively [52]. A systematic review that included 200 patients showed that SVF can be used as a complementary treatment for patients who have failed conservative and arthroscopic treatments, and can be used as a combination of ADSCs, PRP, etc. [53]. Another study compared the effects of different doses (2.5 × 10^7^ versus 5.0 × 10^7^ cells) of SVF in the treatment of OA and showed that the total KOOS and symptom scores of the high-dose group were significantly better than those of the low-dose group at 12 months postoperatively [54]. However, at a mid-term follow-up, a significant improvement in function within 2 years after SVF injection was found, but this effect began to diminish by the third year [55]. Of interest, Rogers et al. produced SVF (PSG-01) for the first time with U.S. Food and Drug Administration (FDA) approval and through Good Manufacturing Practices (GMP), and conducted a phase 1/2A prospective clinical study (NCT 04043819) in OA patients with KL grades of II-IV. The results confirmed that a single intrathecal injection of SVF into the knee joint was not only safe and reliable, but also significantly improved pain and function [56]. Others have mechanically obtained SVF with a viable cell percentage of 64.43%, and 33 patients who received an intra-articular injection of SVF into the knee joint showed significant improvement in VAS scores and the KOOS after 12 months [57]. Similarly, Labarre et al. obtained SVF by mechanical treatment and injected it into the infrapatellar (Hoffa) fat pad. At a 2-year follow-up, it was found that site-specific injections of SVF also improved pain and function in patients with severe OA [58]. A recent meta-analysis showed that SVF significantly improved pain and function in patients with OA of the knee compared to saline and hyaluronic acid, but the short-term advantage over corticosteroids was not significant [59]. In the short term, SVF has also been shown to be effective in improving pain and function in patients with hip OA, and is particularly effective in KL grade II patients [60]. Overall, SVF demonstrated some effectiveness and safety in the short-term treatment of OA. It is necessary for future studies to explore how to prolong the efficacy of SVF and elucidate its mechanism of action in larger randomised controlled trials and with longer-term follow-up, with a view to achieving more durable and stable clinical benefits in the treatment of OA.

## 4. Dedifferentiated Adipocytes in OA

**Figure 2:**
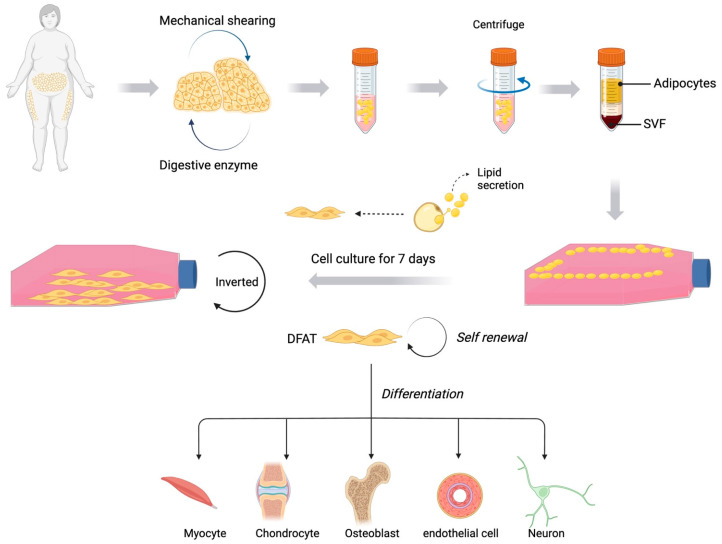
DFAT’s production and use. Adipose tissue is treated mechanically and with digestive enzymes. Mature adipocytes are then separated by centrifugation and transferred to culture flasks filled with culture medium. At first, they are in the upper layer of the culture medium due to their buoyancy. Over time, the adipocytes lose their lipids and transform into DFAT cells with the shape of fibroblasts. The flasks are then inverted to continue the culture of DFAT. DFAT cells are widely used in regenerative medicine and tissue engineering, providing a new approach to tissues such as muscle, bone, cartilage and vascular endothelium, and nerve repair.

In 1986, Sugihara et al. first proposed a technique called ‘ceiling culture’ to obtain DFAT cells in mature adipose tissue [71]. In 2008, Matsumoto et al. found that DFAT has the ability to differentiate into a variety of mesenchymal cell lineages similar to ADSCs [72]. Adipose tissue was digested with digestive enzymes and then the adipocytes were placed in culture medium. Due to their buoyancy, mature adipocytes floated in the medium and, over time, the adipocytes lost significant amounts of lipid (Figure 2). As a result, the adipocytes became more elongated in shape and eventually transformed into a fibroblast-like cell morphology [73,74]. In addition, some studies have found that adipose dedifferentiation can also be induced by modulating TGF-β1 signalling followed by the induction of related genes or by altering the extracellular osmotic pressure [75,76]. DFAT cells are considered analogues of ADSCs due to their strong proliferative and multidifferentiation capacity [72,77]. Compared to pluripotent stem cells, DFAT has a lower risk of tumourigenesis [78]. In in vitro experiments, DFAT can differentiate into various cell types such as chondrocytes, osteoblasts, myocytes, and vascular endothelial cells [79,80,81]. In a rat cartilage defect model, DFAT-derived cell microcolonies significantly promoted cartilage repair [77]. A follow-up study further demonstrated that DFAT could highly express genes related to cartilage protection (e.g., Proteoglycan 4 (PRG4) and Bone morphogenetic protein 6 (BMP6)) and could inhibit the expression of ADAMTS4 and IL6 when stimulated by inflammatory factors [82]. In another study, infrapatellar fat pad-DFAT was shown to be superior to infrapatellar fat pad adipose stromal cells in terms of the chondrogenic capacity [83]. Table 3 presents information about DFAT’s progress in the field of regenerative medicine.

Although existing evidence shows that DFAT has potential value in cartilage regeneration and inflammation modulation, its biosafety and cell purity need to be studied in depth and its clinical application is still in the early exploratory stage [74]. As the safety and mechanism of DFAT are further studied in the future, the therapeutic potential of DFAT in degenerative diseases such as OA is still worthy of investigation.

## 5. Adipose Tissue-Derived Stem Cells in OA

Mesenchymal stem cells (MSCs) have been widely shown to reduce inflammatory responses in the arthritic setting [104,105,106,107,108]. In addition to bone marrow and adipose tissue, MSCs can also originate from a variety of human tissues such as neonatal cord blood and placenta [109,110,111,112]. However, the number of ADSCs in an equal volume of adipose tissue and bone marrow tissue is approximately 500 times that of bone marrow MSCs [113,114]. In a meta-analysis, adipose-derived MSCs were shown to be superior to bone marrow-derived MSCs in terms of safety and ability to improve function [115]. ADSCs have received increasing attention as an easily accessible and abundant pluripotent stem cell resource [116,117].

ADSCs have been shown in several animal studies to play an important role in synovial and cartilage protection. For example, ADSCs can inhibit synovial inflammation by downregulating S100A8/A9 and P2 × 7 receptors and reduce the expression of matrix metalloproteinase 13 (MMP13), thereby slowing down cartilage degradation [118,119,120]. Xu et al. found that ADSCs inhibited the TNF-α-induced chondrocyte pyroptosis signalling pathway [121]. Recent studies have also indicated that upregulation of IL-6 in ADSCs enhances immunomodulation and inhibits nuclear factor κ-Β receptor activator ligand (RANKL), thereby reducing cartilage degeneration [122].

In terms of clinical studies, a significant improvement in VAS scores and KOOS was found in 42 patients who received intra-articular injections of ADSCs, and an increase in the injection dose did not provide a benefit at a 12-month postoperative follow-up [67]. Hosono et al. also stated that multiple injections of ADSCs may present with severe arthritis and the aberrant expression of histone H2B antibodies [123]. Another study reported that those patients who underwent medial open wedge high tibial osteotomy (HTO) followed by articular injection of ADSCs observed better cartilage regeneration by arthroscopy 2 years later [124]. Hatano et al. found that intra-articular ADSC injections improved hip function within 6 months. Female patients and patients with moderate hip OA showed better outcomes [125].

An allogeneic human ADSC product (ELIXCYTE ^®^) showed a favourable safety and tolerability profile in a Phase I/II OA clinical trial (NCT02784964) [126]. It was also found to control the progression of OA by lowering MMP13 and increasing levels of anti-inflammatory cytokines (IL-1RA, IL-10, and IL-13) in a follow-up study [127]. In conclusion, ADSCs show promising opportunities for applications in tissue repair and inflammation regulation in OA, and more large-scale clinical trials and in-depth mechanistic studies are needed to optimise their efficacy and safety in the future.

## 6. Adipose-Derived Stem Cell Extracellular Vesicles in OA

**Figure 3:**
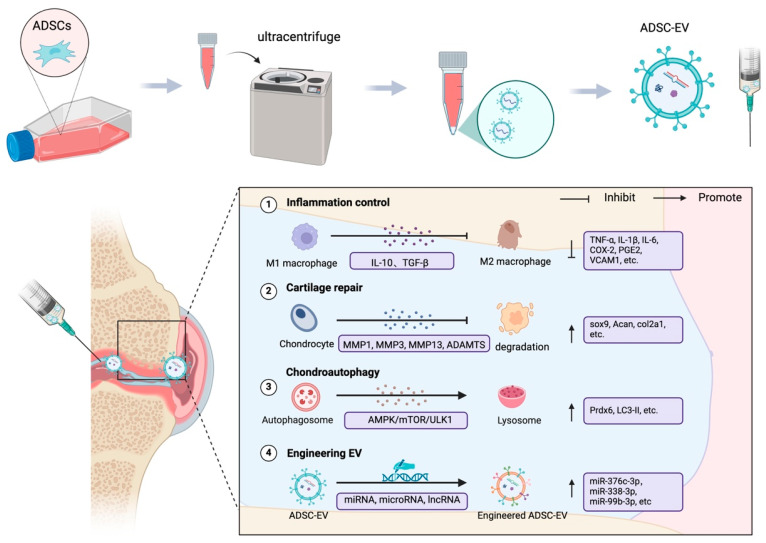
Isolation, design, and therapeutic efficacy of ADSC-EVs in OA treatment: ADSC-EVs were obtained by ultracentrifugation, and these EVs have multiple therapeutic effects on cartilage regeneration. First, they modulate macrophage polarisation by promoting IL10 and TGF- β and inhibit pro-inflammatory mediators such as TNF-α, IL-1β, and IL-6. Second, ADSC-EVs support cartilage repair by inhibiting catabolic enzymes (MMP1, MMP3, MMP13, and ADAMTS, etc.) and enhancing the production of extracellular matrix components (including Sox9, Acan, and Col2a1) to support cartilage repair. In addition, they regulate chondrocyte autophagy and promote intracellular homeostasis through pathways such as AMPK/mTOR/ULK1. ADSC-EVs can be engineered to enhance their therapeutic potential.

In 1981, Trams et al. proposed that cells can secrete nanoscale vesicles with biological functions and introduced the concept of ‘exosomes’ [128]. However, the latest guidelines of the International Society for Extracellular Vesicles (ISEV) from 2023 recommend the use of ‘extracellular vesicles’, avoiding terms based on biogenesis such as ‘exosome’ [129]. Therefore, in this article, EVs are used as a blanket term. ADSC-EVs play a key role in mediating therapeutic effects such as angiogenesis, inflammation control, and tissue repair [130,131,132]. EVs are less immunogenic, have a lower risk of embolisation, and are more stable than cell-based therapies [133,134,135] (Figure 3) (Table 4).

### 6.1. ADSC-EVs and the Regulation of Inflammation in OA

Pro-inflammatory cytokines such as interleukin-1β (IL-1β) and tumour necrosis factor-α (TNF-α) play a key role in the pathological process of OA [136,137]. Meanwhile, IL-1 and TNF-α also induce cyclooxygenase-2 (COX-2) and increase the release of prostaglandin E2 (PGE2), further exacerbating the inflammatory response [138]. As a result, there has been a proliferation of studies that demonstrate inhibition of the inflammatory response to improve OA. Liu et al. used anion-exchange chromatography to isolate EVs from infrapatellar fat pad-derived MSCs, which were injected into the joints of OA mouse models. The levels of pro-inflammatory cytokines in chondrocytes were found to be significantly reduced and controlled the progression of OA [139]. ADSC-EVs treated with synovial fluid from OA patients secrete miRNAs with chondroprotective functions (miR-193b-3p, miR-24-3p, and miR-92a-3p with miR-21-5p) [140]. Notably, an appropriate hypoxic microenvironment is beneficial to MSC function [141]. Correspondingly, hypoxia-induced ADSC-EVs slowed down OA progression by suppressing the senescence-associated secretory phenotype (SASP) [142]. ADSC-EVs pretreated with IFN γ secreted miRNA to upregulate the M2 macrophage marker CD163, and downregulated the chondrocyte inflammation marker Vascular cell adhesion molecule 1 (VCAM1) [143]. ADSC-EVs also inhibit M1 macrophages in the synovium and downregulate A disintegrin and metalloproteinase with thrombospondin motifs 9 (ADAMTS9), which further suppresses inflammation and alleviates OA progression [144,145].

### 6.2. ADSC-EVs Promote Cartilage Repair

The role of ADSC-EVs in OA cartilage repair has also attracted much attention (Figure 3). Zhao et al. found that ADSC-EVs upregulated miR-145 and miR-221 in chondrocytes, thereby enhancing cartilage formation [146]. Tofiño-Vian et al. demonstrated that ADSC-EVs reduced matrix metalloproteinase (MMP) activity and MMP-13 release, while promoting the production of type II collagen and anti-inflammatory factors (e.g., IL-10) [147]. By co-culturing ADSC-EVs with a cartilage injury model, sox9, hyaline cartilage-specific gene aggrecan (Acan), and col2a1 protein secretion could be promoted, contributing to cartilage regenerative effects [148].

### 6.3. Promotion of Chondrocyte Autophagy

In the pathological setting of OA, the level of autophagy in chondrocytes is generally reduced, especially in aged chondrocytes [149,150]. This reduced autophagy is thought to be closely related to chondrocyte death and matrix degeneration. Studies have shown that infrapatellar fat pad-derived EVs can inhibit the mTOR signalling pathway via miR-100-5p to enhance chondrocyte autophagy [151]. ADSC-EVs also upregulate peroxidase 6 (Prdx6) and the autophagy marker LC3B, thereby protecting chondrocytes [152]. Meng et al. found that ADSC-EVs could regulate autophagy and promote chondrocyte repair by promoting FEZ2 through miR-429 in an OA mouse model [153].

### 6.4. Engineering ADSC-EVs

To further enhance the therapeutic potential of ADSC-EVs, studies have focused on their genetic or material modification in recent years (Figure 3). By upregulating miR-376c-3p, the WNT-β-catenin signalling pathway could be inhibited to alleviate chondrocyte apoptosis and synovial fibrosis [154]. Zhao et al. effectively ameliorated the pathological changes in cartilage by engineering subcutaneous adipose ADSC-EVs with targeted delivery of miR-199a-3p [155]. Li et al. used miR-338-3p to modify ADSC-EVs to target RUNX2 expression to inhibit chondrocyte inflammation and degradation [156]. It has also been shown that the miR-99b-3p modification of ADSC-EVs followed by hydrogel particle (HMP) encapsulation could achieve sustained local drug release for OA [157]. By culturing ADSCs on hyaluronic acid (HA)-coated surfaces, CD44-enriched ADSC-EVs could be obtained, which could downregulate pro-inflammatory cytokine and chemokine expression in OA models [158]. Combining ADSC-EVs with 3D-printed specific biomimetic hydrogel scaffolds effectively promoted cartilage regeneration in a rat model [159]. A recent study also found that TNF-α pretreatment of infrapatellar fat pad-derived EVs activated the PI3K/AKT signalling pathway and increased the secretion of EVs, while ameliorating the arthropathological changes in OA mice [160]. As a novel cell-free therapeutic tool, ADSC-EVs not only demonstrated significant advantages in the modulation of inflammation and cartilage repair in the pathological setting of OA, but also could be engineered to further enhance targeting and therapeutic potential. In the future, large-scale randomised controlled clinical trials and in-depth mechanistic studies will help to clarify the optimal preparation process, dosage, and administration timing of ADSC-EVs, and provide safer, more effective and personalised precision treatment options for OA.

**Table 4:** ADSC-EVs and OA research.

Time	Model	Moulding Method	Mechanism	Results	Reference
2017	OA osteoblasts	IL-1β	↓ HNE-modified proteins ↑ mitochondrial membrane potential	↓ ageing-related β-galactosidase activity and γ H2AX, IL-6, PGE 2	[161]
2018	OA chondrocytes	IL-1β	↓ NF-κB and activator protein-1 ↑ membrane-bound protein A1	↓ PGE 2, MMP-13 ↑ IL-10, collagen II	[147]
2020	OA chondrocytes; rats	IL-1β, MIA, DMM	-	↑ chondrocyte proliferation and migration, collagen II ↓ MMP-1, MMP-3, MMP-13, and ADAMTS-5, M1 Macrophage infiltration	[144]
2020	OA chondrocytes, synovial fibroblasts, periosteal cells	H_2_O_2_	↑ miR-145, miR-221 Wnt/β-catenin pathway	↓ IL-6, NF-κB, TNF-α ↑ IL-10, Collagen II and β-catenin	[146]
2021	OA chondrocytes, synovial cells	IL-1β	↓ NF-κB pathway	↓ IL-6, IL-8, MCP-1, MMP-1, MMP-10 and ADAMTS5	[162]
2021	OA chondrocytes	IL-1β	Peroxidase 6	↓ IL-6, MMP-13 ↑ autophagy protein LC3B	[152]
2022	OA chondrocytes, synovial cell	IL-1β	↓ NF-κB pathway	↓ IL-6, IL-8, MCP-1, COX-2 and VEGF, MMP-1, MMP-13 and ADAMTS-4, TNF-α	[163]
2022	OA chondrocytes, synovial cell, mice	IL-1β, DMM	↓ endoplasmic reticulum stress (miR-486-5p)	↓M1 macrophage, IL-6, TNF-α, MMP13 ↓ CHOP, Caspase-3 and GRP78 ↑ collagen II	[164]
2022	OA chondrocytes, synovial cells, rats	IL-1β, lipopolysaccharide, MIA	Targets the WNT-β-catenin pathway (MicroRNA-376c-3p)	↑ collagen II, β-catenin, Aggrecan ↓ TNF-α and IL-6, IL-1β, IFN-γ, α-SMA and collagen III, MMP13, ADAMTS5	[154]
2023	Rats	IL-1β	Targets ADAMTS9 to activate the PI3K/AKT/mTOR pathway (miR-93-5p)	↓ IL-6, IL-1β, TNF-α, and iNOS	[145]
2023	Primary articular chondrocytes, rats	IL-1β, ACLT	Protoelastin induces miR-451-5p	↑ collagen II and SOX 9; cartilage ECM ↑ OARSI and Mankin scores	[165]
2023	Primary articular chondrocytes, rats	IL-1β, MIA	miR-429 targets FEZ2	↑ chondrocyte autophagy	[153]
2023	Primary articular chondrocytes, rats	IL-1β, ACLT	Hypoxia inhibits SASP secretion	↓ ADAMTS5, MMP13, IL-6, and TNF-α ↑ proteoglycans and collagen II	[142]

ACLT: anterior cruciate ligament severance; SASP: senescence-associated secretory phenotype, DMM: destabilisation of the medial meniscus, MIA: monosodium iodoacetate; IL, interleukin; HNE, hydroxynonenal; NF-κB, Nuclear factor kappa-light-chain-enhancer of activated B cells; MCP-1, Monocyte Chemoattractant Protein-1; COX-2, Cyclooxygenase-2; VEGF, Vascular endothelial growth factor; γ-H2AX, γ-Hydroxybutyric acid; PGE 2, Prostaglandin E2; MMP, matrix metalloproteinase; ADAMTS5, A disintegrin and metalloproteinase with thrombospondin motifs 5; TNF-α, tumour necrosis factor-α, CHOP, C/EBP homologous protein; GRP78, 78 kDa glucose-regulated protein; IFN-γ, interferon-γ; α-SMA, α-smooth muscle actin; iNOS, inducible nitric oxide synthase; SOX, Sex-determining region Y -box; ECM, Cartilage extracellular matrix; OARSI, Osteoarthritis Research Society International; FEZ2, Fasciculation And Elongation Protein Zeta 2; SASP; senescence-associated secretory phenotype.

## 7. Cell-Free Fat Extracts for OA

**Figure 4:**
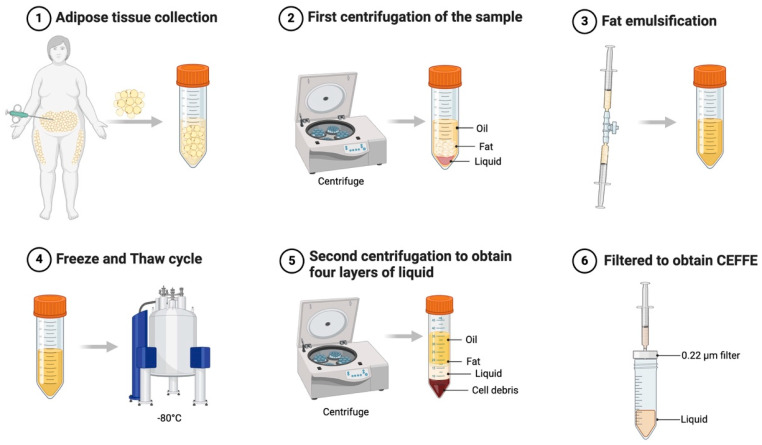
Preparation process of CEFFE from adipose tissue: (**1**) Adipose tissue is collected through surgical excision or liposuction. (**2**) The sample undergoes the first centrifugation, separating it into oil, fat, and liquid layers. (**3**) The fat is emulsified using mechanical shear forces. (**4**) A freeze–thaw cycle at −80 °C disrupts cell membranes, releasing bioactive factors. (**5**) A second centrifugation further separates four distinct layers: oil, fat, liquid, and cell debris. (**6**) The liquid phase is filtered through a 0.22 µm membrane to obtain CEFFE.

To prepare CEFFE, adipose tissue obtained after liposuction was washed with saline, centrifuged, mechanically emulsified, chilled at −80 °C, cryocycled, and subsequently re-centrifuged to obtain four layers of liquid (Figure 4). A cell-free aqueous extract was obtained by taking the third layer of the liquid and filtering it through a 0.22 μm filter to remove the cellular components [166]. In a mouse model of ischaemia, CEFFE was found to contain several growth factors (e.g., BDNF, GDNF, TGF-β, etc.) and significantly promote angiogenesis [166]. In recent years, studies have further demonstrated that CEFFE accelerates angiogenesis, thereby improving flap survival and the quality of expanded skin [167,168]. It also improves fertility by increasing anti-Müllerian hormone, estradiol (E2), and follicle-stimulating hormone levels in ovarian insufficiency (POI) mice [169]. In addition, CEFFE can increase the number of CD31-positive capillaries and Ki67-positive cells in a mouse model of androgenetic alopecia, thereby reducing hair loss [170].

In terms of OA treatment, Jia et al. found that CEFFE upregulated the proportion of CD206^+^ macrophages in the synovial membrane in an OA rat model experiment. In in vitro experiments, CEFFE inhibited the expression of interleukin 6 (IL-6) and A disintegrin and metalloproteinase with thrombospondin motifs 5 (ADAMTS-5) in chondrocytes, while upregulating SOX-9 [17]. In further studies, it was shown that membrane-linked protein (Annexin) A5 in CEFFE inhibited M1-type macrophage polarisation by promoting the endocytosis and degradation of toll-like receptor (TLR) 4, which reduced inflammatory factor levels and protected chondrocytes, and that Annexin A5 significantly relieved joint pain and attenuated cartilage damage in an animal model [171]. Taken together, these findings provide new potential directions for the application of CEFFE and its active ingredients in OA therapy.

## 8. Controversies and Challenges

As adipose tissue derivatives are increasingly studied in the treatment of OA, their clinical translation still faces many controversies and challenges. Firstly, there is no consensus on the need for enzymatic digestion of adipose tissue: mechanical treatment often makes it difficult to sufficiently disrupt the extracellular matrix, resulting in a large number of cells trapped in tissue debris [172]. In contrast, enzymatic digestion yields higher numbers of progenitor cells, but at the same time, has the potential to impair the functional expression of ADSCs [173,174]. Although enzymatic digestion is approximately 1000-fold more efficient than mechanical processing [175,176], mechanical processing of adipose tissue is shorter and more cost-effective [19,177]. There are no globally harmonised standards of operation. Preparation methods vary greatly from organisation to organisation, resulting in inconsistent cell activity, purity, content, and safety results.

In practice, there are multiple operational steps whether enzymatic digestion or mechanical separation is used, potentially increasing the risk of infection. In order to reduce contamination, studies have attempted to use techniques such as closed mechanical processing systems and three-step collection systems [178]. In addition, some studies have suggested that stem cells may be precursor cells for solid tumours due to their vasculogenic and immunomodulatory functions [179,180]. ADSCs have been found to be potential initiators of synovial sarcoma [181]. Koellensperger et al. have observed that ADSCs enhance gene expression and angiogenesis in breast cancer cells in vitro [182]. However, it has also been found that stem cells cultured for a long period of time may undergo genomic alterations but do not cause cancer [183,184]. Although no clinical trials have reported malignant transformations of ADSCs, the long-term safety of ADSCs should be continuously monitored with genetic safety testing and follow-up. In addition, due to the vascularisation and osteogenic potential of ADSCs, their intra-articular use may induce ectopic osteogenesis or chondrogenesis, which may interfere with the original joint structure, and even cause pain or osteophytes. The above controversies and potential risks highlight the need for further in-depth research and standardisation in the treatment of OA with fat derivatives.

## 9. Regulations and Limitations

Currently, no products related to EVs have been approved worldwide for any use, and the FDA classifies exosomes as biological products [185]. Therefore, safety and efficacy verification of the product is required. The European Medicines Agency (EMA) considers any substantially manipulated cell therapy such as adipose stem cells (ASCs) to be an advanced therapeutic medicine and is therefore closely regulated [186]. SVF involves mechanical or enzymatic processing, and it is difficult to distinguish between minimal and advanced manipulation. As a result, SVF products are often categorised as requiring high standards of approval, and the FDA has also classified SVF as a biological product requiring clinical trials (Product 351) [187]. This means that SVF products must undergo the same level of safety and efficacy clinical trials as new drugs, which is a complex and costly process.

## 10. Current Status and Future Perspectives

Adipose tissue-derived therapies are increasingly used in the treatment of OA, but there is still a lack of uniformity in their efficacy. Nguyen et al. recently compared the efficacy of ADSCs and SVF in 452 patients with OA. They found that ADSCs provided longer-lasting pain relief, while SVF showed a faster onset of action. ADSCs were also more effective in promoting cartilage regeneration. The authors suggested that SVF may be more suitable for older patients or those with a BMI greater than 30 [188]. However, another meta-analysis reported that autologous cultured ADSCs began to relieve pain at around 3 months post-treatment, while SVF took up to 12 months to show significant effects [189]. Maeda et al. conducted a retrospective study of 72 patients with OA treated with either SVF or MFAT. They found that MFAT treatment resulted in greater improvements in knee flexion and cartilage quality, while SVF treatment resulted in faster pain relief [190]. Some meta-analyses of RCTs showed that ADSCs and SVF had a similar short-term efficacy [191,192]. Another meta-analysis, which included 79 RCTs, found that SVF was more effective than other adipose-derived therapies such as MSCs in reducing pain and improving joint function [193]. Studies comparing CEFFE and ADSC-EVs with other adipose tissue derivatives are still limited. However, available studies suggest that CEFFE may be superior to hyaluronic acid in reducing joint inflammation and improving chondrocyte metabolism [194]. Although high-quality evidence for non-cellular therapies is still lacking, future clinical studies are expected to provide further validation.

With the development of technology, new methods like 3D printing and bioscaffolds are improving adipose-derived therapies for OA. Nonaka et al. used 3D printing to assist ADSC chondrogenesis, and the resulting cartilage showed properties similar to those of normal cartilage [195]. Gelatin microcarriers were also used to culture and deliver ADSCs, which increased EV secretion and reduced the friction coefficient at the cartilage surface [196]. In another study, ADSCs were combined with bioporous scaffolds to release transforming growth factors (e.g., TGF-β1), thereby promoting cartilage regeneration [197]. Meanwhile, a hybrid scaffold (Gel-DCM) formed by combining photoreactive gelatin hyaluronic acid hydrogel (Gel) with directed porous decellularised cartilage matrix (DCM) provided a 3D microenvironment for ADSCs, which further promoted their cartilage differentiation [198]. Three-dimensional β-tricalcium phosphate (β-TCP) bioceramic scaffolds combined with ADSCs have also demonstrated good mechanical support and form a thicker cartilage layer [199]. The combination of emerging technologies with adipose tissue-derived therapies demonstrates great potential to promote cartilage repair and improve osteoarthritis outcomes.

Non-cellular products, such as EVs and cell-free extracts, are increasingly gaining prominence in adipose-derived therapies. These strategies are able to avoid the potential risk of cancer development or ectopic osteogenesis in live cell therapy, while demonstrating multiple therapeutic efficacies in repairing cartilage, modulating joint inflammation, and promoting angiogenesis. Future research should prioritise the establishment of uniform extraction and quality control standards to ensure the consistency and reproducibility of the active molecule content and biological effects.

## 11. Conclusions

Fat derivatives show strong potential in the treatment of OA. Different forms of fat sources can help reduce pain in patients and improve joint function through multiple pathways, including modulating the inflammatory microenvironment and promoting chondrocyte repair and angiogenesis. Compared with traditional drugs or surgery, fat derivatives are highly regarded for their advantages of easy access, simple handling, and relatively low ethical risks. However, there is a lack of uniform preparation process and quality control standards, and safety issues such as ectopic osteogenesis and potential tumour risk have not been fully clarified. With the deepening of multidisciplinary research and the continuous optimisation of key technologies, fat derivatives are expected to provide a more precise, efficient, and personalised regenerative medicine option for OA.

## Figures and Tables

**Table 2:** Clinical studies of SVF in the treatment of OA.

Time	Country	Patient Number	Position (KL)	Age (Mean, Years)	Follow-Up	Results	Complications	Reference
2017	Japan	13 (2M/11F)	Knee OA (II-IV)	74.5 ± 5.4	6 months	↓ WOMAC score (%), VAS	Pain and swelling in injection and fat harvesting sites	[61]
2018	China	16 (3M/13F)	Knee OA (II-IV)	53 ± 10.97 (left) 51 ± 5.95 (right)	12 months	↓ VAS, WOMAC score (%) ↑ ROM	Pain in fat harvesting site (25% of patients) Post-injection pain (37.5% of patients)	[51]
2019	Italy	20 (9M/11F)	Knee OA (I-IV)	59.6 ± 10.5	18 months	↓ VAS, WOMAC score (%)	One case of occult swelling in the suprapatellar region Most patients felt “tied knee”	[62]
2019	Croatia	20 (15M/5F)	Knee OA (III-IV)	40–85	12 months	↓ VAS, WOMAC score (%) ↑ KOOS	None	[63]
2019	Japan	38 (7M/31F)	Knee OA (II-IV)	73 ± 9.1	6 months	↓ VAS; ↑ KOOS	Knee joint effusion after injection (8% of patients) Minor complications in fat harvesting site (34% of patients)	[15]
2020	America	39 (17M/22F)	Knee OA (II-III)	59.0 ± 9.9	12 months	↑ WOMAC score (%)	Post-injection pain and swelling (1 patient)	[64]
2020	Japan	57 (41M/16F)	Knee OA (II-IV)	69.4 ± 6.9	12 months	↓ VAS, WOMAC score (%) and ↑ KOOS ↑ MRI: T2 mapping values of lateral femur and tibia ↑ knee extension angle	None	[52]
2020	Germany	12 (7M/5F)	Knee OA (III-IV)	61 (51–80)	12 months	↑ KOOS; subjective satisfaction: 67%	Post-injection painless swelling Haematoma and muscle soreness in fat harvesting site	[65]
2022	China	47 (18M/29F)	Knee OA (II-III)	50.83 ± 10.88	12 months	↓ cartilage defect thickness, VAS, WOMAC score (%)	Post-injection pain and swelling	[66]
2022	Germany	33 (18M/15F)	Knee OA (I-IV)	60.58(23–88)	12 months	↓ VAS and ↑ KOOS ↑ VR12 psychological scores	None	[57]
2022	Japan	38 (18% M/82% F)	Knee OA (II-IV)	73.6 ± 9	24 months	↓ VAS and ↑ KOOS	Pain, bleeding, induration in fat harvesting site (13 patients) Post-injection pain and swelling (3 knees)	[67]
2022	Russia	16 (7M/9F)	Knee OA (II-IV)	61 (57–64)	12 months	↓ VAS and ↑ KOOS	Almost all patients had post-injection site discomfort; some reported painless swelling at the injection site A proportion of 27% of patients had a slight increase in temperature (37.2–37.6 °C)	[68]
2023	India	58	Knee OA (I-III)	45–85	36 months	↓ VAS and ↑ KOOS	Pain, swelling, and bruising in fat harvesting site (some patients)	[69]
2023	Japan	Single injection group: 30 (8M/22F) Double injection group: 24 (6M/18F)	Knee OA (II-IV)	Single injection group: 68.8 ± 8.2 Double injection group: 69.1 ± 11.8	24 months	↓ WOMAC score ↑ HKA angle and the mean T2 mapping values	Post-injection pain and swelling 9.3% (single injection group) and 8.3% (double injection group)	[70]
2023	Japan	42 (5M/37F)	Hip OA (II-IV)	60.2 ± 9.4	6 months	↑ HHS, JHEQ scores and ↓ VAS	Mild hip pain (5 patients (11.9%))	[60]
2024	USA	29 (9M/20F)	Knee OA (II-IV)	65.6	12 months	↑ KOOS	Mild to moderate post-injection pain or itching (6 patients) Mild to moderate pain, bruising, subcutaneous haematoma or numbness in fat harvesting site (17 patients)	[56]
2025	Germany	25 (14M/11F)	Knee OA (IV)	53–67	24 months	↓ VAS and ↑ KOOS, ADL and QOL scores	None	[58]

M: male; F: female; KL: Kellgren–Lawrence; VAS: visual analogue scale; KOOS: Knee injury and Osteoarthritis Outcome Score; WOMAC: Western Ontario and McMaster Universities Osteoarthritis Index; DASH: Disabilities of the Arm and Shoulder; PRWE: Patient Wrist Evaluation; ROM: Range of Motion; HKA: Hip–Knee–Ankle; HHS: Harris Hip Score; JHEQ: Japanese Orthopaedic Association Hip Disease Evaluation Questionnaire; ADL: Activity of Daily Living; QOL: quality of life.

**Table 3:** DFAT and regenerative medicine research.

Time	Disease	Animal	Results	Reference
2008	Chronic renal dysfunction	Mice	Improvement of glomerulosclerosis, ↓ TGF-β1 and fibronectin mRNA in renal cortex ↓ serum BUN	[84]
2008	Spinal cord injury	Rat	↑ βIII microtubule protein; BDNF; GDNF	[85]
2012	Infarcted myocardium	Mice	↑ endothelial cells	[81]
2014	Periodontal tissue loss	Rat	↑ proliferating cell nuclear antigen; periodontal tissue regeneration	[86]
2014	Spinal cord injury	Mice	↑ motor function of the hind limbs; neurotrophic factor; astrocytes and oligodendrocytes	[87]
2015	Artificial dermal graft	Rat	↑ capillary infiltration; endothelial cells; thickness of dermal-like tissue	[88]
2015	Light-aged	Mice	↑ TGF-β1; collagen I and III ↓ MMP-1 and MMP-3	[89]
2015	Glomerulonephritis	Rat	↑ TSG-6 ↓ macrophage infiltration and IL-6, IL-10, and IL-12β	[90]
2016	Vesicoureteral reflux	Rat	↓ ureteral internal/external diameter ratio and connective tissue area in the posterior bladder wall ↓ apoptosis of renal pelvic urinary tract epithelial cells	[91]
2017	Hypoxic–ischemic encephalopathy	Rat	↓ brain cell death rate	[92]
2018	Cerebral infarction	Mice	↑ Nestin and SOX2; functional recovery	[93]
2018	Knee cartilage defect	Rat	↑ Sox9; collagen II (COL2A1) ↑ ICRS score; modified O’Driscol histological score	[77]
2019	Facial nerve defects	Rat	↑ number of myelinated fibres; thickness of myelin sheaths in the spinal cord	[94]
2019	Mandibular bone defect	Rat	↑ bone regenerationd; bone width	[95]
2020	Inflammatory bowel disease	Mice	↑ TRAIL, IDO1, and NOS2 ↓ T-cell proliferation	[96]
2021	Mandibular defects in osteoporotic	Rat	↑ ERK1/2 and Smad2 phosphorylation signalling pathways	[97]
2022	Intra-periodontal bone defects	Rat	↑ ALP, Runx2, OPN	[98]
2022	Glomerulonephritis	Mice	↑ microRNA 23b-3p; TSG-6 mRNA; PGE2 and IL-10 mRNAs; CCL-17 ↓ CD44 mRNA; TNF-α and MCP-1	[99]
2022	Periodontal Class II bifurcation defects	Small porcine	↑ cytoskeletal, periodontal ligament-like fibres and alveolar bone formation	[100]
2022	Persistent stress urinary incontinence	Rat	↑ leak point pressure; urethral transverse muscle; smooth muscle	[101]
2023	Neonatal necrotizing enterocolitis	Rat	↓ IL-6; CCL-2	[102]
2024	OA	Rat	↑ PTGS2, TNFAIP6, and BMP2; ↓ ADAMTS4 and IL6 in synovial fibroblasts	[82]
2024	Anorectal sphincter dysfunction	Rat	↑ MyoD and myogenin genes; mature myocytes	[103]

TGF-β1, Transforming growth factor beta 1; BUN, blood urea nitrogen; GDNF, Glial Cell Line-derived Neurotrophic Factor; BDNF, Brain-derived neurotrophic factor; MMP, matrix metalloproteinase; TSG-6, Tumour necrosis factor-inducible gene 6; IL, interleukin; SOX, Sex-determining region Y-box; ICRS, International Cartilage Repair Society; TRAIL, TNF-related apoptosis-inducing ligand; IDO1, Indoleamine 2,3-dioxygenase; NOS2, Nitric oxide synthase 2; ALP, Alkaline phosphatase; Runx2, Runt-related transcription factor 2; OPN, Osteopontin; TNF-α, Tumour necrosis factor-α; MCP-1, Monocyte Chemoattractant Protein-1; CCL, C-C motif chemokine ligand; PTGS2, prostaglandin-endoperoxide synthase 2; TNFAIP6, Tumour Necrosis Factor-Inducible Gene 6 Protein; BMP2, Bone morphogenetic protein 2; ADAMTS4, A disintegrin and metalloproteinase with thrombospondin motifs 4; MyoD, Myoblast determination protein 1.

## Data Availability

Not applicable.
